# Factors influencing therapeutic efficacy of non-lactational mastitis based on hematological inflammatory markers and establishment of a predictive model

**DOI:** 10.3389/fmed.2025.1668664

**Published:** 2026-01-02

**Authors:** Jinjuan Peng, Li Li, Lili Fan, Qun Lu

**Affiliations:** 1 Department of Breast Surgery, The Fourth People’s Hospital of Zhenjiang, The Fourth Affiliated Hospital of Jiangsu University, Zhenjiang, China; 2 Department of Breast Surgery, Changzhou Maternal and Child Health Care Hospital, Changzhou, China

**Keywords:** inflammatory markers, non-lactational mastitis, mastitis, SII, CRP

## Abstract

**Objective:**

To explore factors influencing the therapeutic efficacy of non-lactational mastitis (NLM) based on hematological inflammatory markers and to establish a predictive model.

**Methods:**

Two hundred and sixty-four cases of NLM patients admitted to The Fourth People’s Hospital of Zhenjiang and Changzhou Maternal and Child Health Care Hospital from January 2019 to December 2022 were retrospectively selected. The patients were divided into a modeling group (*n* = 185) and a validation group (*n* = 79) in a 7:3 ratio with a random number table method. In the modeling group, patients were further divided based on therapeutic efficacy into a good efficacy group (*n* = 133) and a poor efficacy group (*n* = 52).

**Results:**

The results of binary logistics regression analysis showed that SII and CRP were influencing factors of therapeutic efficacy of NLM (*p* < 0.05). The formula for the model expression was Logit(P) = −74.457 + (0.823X_1_) + (0.589X_2_). The calibration curve slope of the model in the modeling group and validation group was close to a straight line, indicating good consistency between the predicted risk and actual risk. The ROC analysis results showed that the area under the curve (AUC) of the model in the modeling group was 0.94 (95% CI: 0.823–0.966), the area under the curve of the model in the validation group was 0.85 (95% CI: 0.794–0.893), drawing DCA curves to evaluate the clinical utility of the model in predicting efficacy showed clear positive net benefits, indicating good clinical utility.

**Conclusion:**

SII and CRP are influencing factors of therapeutic efficacy of NLM. This study successfully established a prediction model based on hematological inflammatory markers.

## Highlights

- This study investigated the influencing factors of therapeutic efficacy in non-lactational mastitis and established a predictive model based on hematological inflammatory indicators to enhance the accuracy of clinical decision-making.
- Overweight/obesity, SII, and CRP are associated with the therapeutic efficacy of non-lactational mastitis. Binary logistics regression analysis confirmed SII and CRP as independent influencing factors of efficacy.
- A predictive model based on SII and CRP was developed, with the model expression as Logit(P) = −74.457 + (0.823X_1_) + (0.589X_2_).
- The predictive model established in this study exhibits good clinical utility and accuracy, providing robust support for the treatment of non-lactational mastitis.

## 1 Introduction

Non-lactational mastitis (NLM) is a complex and challenging breast disease that significantly impacts the physical and mental health of women (1). Its incidence is steadily increasing. In recent years, the number of NLM cases has been on the rise globally, accounting for 0.3 to 1.9% of all breast diseases worldwide. In China, this proportion is even higher, ranging from 2 to 5%, indicating a relatively high incidence of this disease in breast disorders in China (2). Due to the unclear etiology of the disease, diverse clinical presentations, and lack of specific symptoms, diagnosis is challenging and often prone to misdiagnosis, posing significant treatment challenges. Previous studies have shown (1, 3) that hematological inflammatory markers have significant observational value in mastitis, but there are limited researches on their impacts in NLM patients. Therefore, exploring the factors influencing the therapeutic efficacy of NLM based on hematological inflammatory markers and establishing an effective prediction model is of great importance in guiding clinical diagnosis and treatment to improve treatment outcomes (4, 5). This study aims to collect and analyze hematological inflammatory marker data from NLM patients, investigate the relationship between these markers and disease efficacy, and establish a prediction model based on this data to provide personalized treatment plans and prognosis assessments for patients. By in-depth analysis of the changes in inflammatory markers, a better understanding of the pathogenesis of NLM can be achieved, key factors influencing efficacy can be identified, and scientific evidence can be provided for clinical decision-making. Additionally, establishing a prediction model can enhance the accuracy of NLM diagnosis, aid in early identification of high-risk patients, facilitate more proactive and effective treatment measures, reduce disease recurrence and complication rates, and improve patients’ quality of life.

## 2 Research objects and methods

### 2.1 Research objects

**Figure 1:**
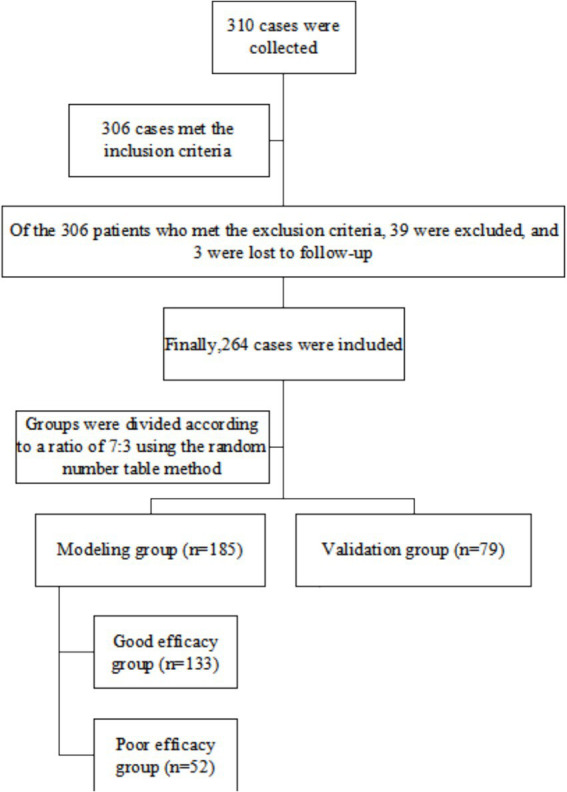
Flowchart.

Two hundred and sixty-four cases of NLM patients admitted to The Fourth People’s Hospital of Zhenjiang and Changzhou Maternal and Child Health Care Hospital between January 2019 and December 2022 were retrospectively selected. Patients were divided into a modeling group (*n* = 185) and a validation group (*n* = 79) in a 7:3 ratio with a random number table method. Within the modeling group, patients were further categorized based on therapeutic efficacy into a good efficacy group (*n* = 133) and a poor efficacy group (*n* = 52). The grouping process is illustrated in Figure 1. The inclusion criteria were as follows: (1) meeting the diagnostic criteria for NLM patients (6); (2) aged between 18 and 60 years; (3) patients with complete clinical follow-up data. The exclusion criteria were as follows: (1) presence of malignant tumors; (2) presence of breast tuberculosis or specific granulomatous lesions (such as nodular disease, fungal infections, Wegner granulomatosis, etc.); (3) loss to follow-up due to other reasons; (4) The patient has other diseases that affect blood inflammatory factors, such as pneumonia, respiratory diseases, urinary tract infections, rheumatoid arthritis, lupus, etc.; (5) Patient had abscesses.

### 2.2 Research methods

#### 2.2.1 Evaluation of therapeutic efficacy

Follow-up for 2 years, the good efficacy group includes patients with significantly relieved symptoms after treatment and no recurrence. The poor efficacy group includes all patients with recurrence (defined as the reappearance of mastitis symptoms within the first year after discontinuing antibiotics for at least 7 days) and those with poor prognosis (7). The poor prognosis criteria are as follows: after standardized treatment, the symptoms of mastitis have not been alleviated or worsened, and local redness, swelling, hot and painful pain persist; an abscess appears in the breast and continues to occur after multiple puncture and drainage or incision and drainage; symptoms of systemic infection are caused, such as fever, chills, fatigue, etc., which affects the patient’s normal life and health.

#### 2.2.2 Criteria for indicators

Overweight/Obesity: body mass index (BMI) ≥25.0 kg/m^2^.

#### 2.2.3 Collection of hematological inflammatory markers

This study was conducted by senior professional laboratory personnel with a solid background in biomedical science and extensive experimental experience. The C-reactive protein (CRP) assay kit (Beijing Lideman Biochemical Co., Ltd., Beijing Medical Device Registration No. 20162400190) was used to measure the concentration levels of C-reactive protein (CRP) in patients’ bodies using the immunoturbidimetric method. Alternatively, magnetic particle chemiluminescence was used. Additionally, the procalcitonin (PCT) detection kit (Nanjing Norman Biological Technology Co., Ltd., Su Jian Zhu Zun 20162401058) was employed to measure PCT levels using a fluorescein-enhanced immunochemiluminescence method, or the PCT detection kit (Zhengzhou Antu Bio-Engineering Co., Ltd., Henan Medical Device Registration No. 20172400746) using magnetic particle chemiluminescence to accurately measure the procalcitonin (PCT) levels in patients’ bodies.

#### 2.2.4 SII calculation

Upon admission, venous blood samples were collected from all subjects to measure neutrophils (NEU), platelet count (PLT), and lymphocytes (LYM) for calculating the Systemic Immune-Inflammation Index (SII) (8) with the formula SII = NEU × PLT/LYM.

### 2.3 Statistical analysis

The experimental data collected were analyzed with SPSS 27.0 (International Business Machines Corporation, Armonk, New York, United States). For normally distributed continuous data in the distribution experiment, values were presented as $\bar{X}\pm S$. Independent sample *t*-tests were used for comparisons. *F*-test was utilized for multiple group comparisons. Count data were expressed as frequencies or rates, and comparisons were made with *χ*^2^ test or Fisher’s exact test. Factors influencing the outcomes were analyzed with both univarite and binary logistics regression analysis. A predictive model was constructed with SPSS. Receiver operating characteristic (ROC) curves were generated with R language, calibration curves were constructed with R language, and decision curve analysis (DCA) curves were used to assess the application value of the model. A significance level of *p* < 0.05 was considered statistically significant for differences.

### 2.4 Quality control

Unified standards were established to clarify the scope, format, and content requirements for data collection. During the collection process, special personnel are arranged to regularly check the integrity of the data, and missing values are promptly traced and supplemented. In the sorting stage, double entry and cross-check methods are used to reduce entry errors. In the verification process, a certain proportion of data is extracted and compared with the original data to ensure accuracy. In addition, regular exchanges and training sessions were organized between personnel from the two institutions to harmonize their understanding of data definitions and standards. These measures ensured that the entire process of data collection, collation, and verification was standardized and rigorous, thereby improving the overall quality of the research.

## 3 Results

### 3.1 General information

Comparison of general information between the modeling group and the validation group showed no statistically significant differences (*p* > 0.05); see Table 1 for details.

**Table 1:** Comparison of general information between modeling group and validation group.

Baseline data		Modeling group (*n* = 185)	Validation group (*n* = 79)	*t*/*χ*^2^ value	*p*-value
Age (years)		35.43 ± 5.41	36.06 ± 5.61	0.857	0.392
Number of pregnancies	<2	106 (57.30)	41 (51.90)	0.654	0.419
	≥2	79 (42.70)	38 (48.10)		
Menstrual disorders	Yes	118 (63.78)	53 (67.09)	0.265	0.607
	No	67 (36.22)	26 (32.91)		
Overweight/Obesity	Yes	58 (31.35)	21 (26.58)	0.600	0.438
	No	127 (68.65)	58 (73.42)		
History of lactational mastitis	Yes	59 (31.89)	26 (32.91)	0.026	0.871
	No	126 (68.11)	53 (67.09)		
History of hyperprolactinemia	Yes	55 (29.73)	25 (31.65)	0.096	0.756
	No	130 (70.27)	54 (68.35)		
History of breast trauma	Yes	35 (18.92)	11 (13.92)	0.960	0.327
	No	150 (81.08)	68 (86.08)		
Abscess formation	Yes	141 (76.22)	63 (79.75)	0.393	0.531
	No	44 (23.78)	16 (20.25)		
Sinus tract formation	Yes	31 (16.76)	15 (18.99)	0.191	0.662
	No	154 (83.24)	64 (81.01)		
History of hormone therapy	Yes	17 (9.19)	8 (10.13)	0.057	0.818
	No	168 (90.81)	71 (89.87)		
SII		154.57 ± 20.54	152.87 ± 21.65	0.606	0.545
PCT (ng/L)		0.60 ± 0.05	0.59 ± 0.05	1.488	0.138
CRP (mg/L)		23.26 ± 4.29	22.25 ± 4.36	1.743	0.082

IL-8, interleukin-8; PCT, procalcitonin; IL-6, interleukin-6; CRP, C-reactive protein.

### 3.2 Univariate analysis of factors influencing therapeutic efficiency of NLM

In the modeling group, comparisons of overweight/obesity, SII, and CRP showed statistically significant differences (*p* < 0.05); see Table 2 for details.

**Table 2:** Univariate analysis of factors influencing therapeutic efficiency of NLM.

Baseline data		Poor efficacy group (*n* = 52)	Good efficacy group (*n* = 133)	*t*/*χ*^2^ Value	*p*-value
Age (years)		35.96 ± 5.46	35.23 ± 5.40	0.824	0.411
Number of pregnancies	<2	31 (59.62)	75 (56.39)	0.690	0.690
	≥2	21 (40.38)	58 (43.61)		
Menstrual disorders	Yes	33 (63.46)	85 (63.91)	0.955	0.955
	No	19 (36.54)	48 (36.09)		
Overweight/Obesity	Yes	22 (42.31)	36 (27.07)	0.045	0.045
	No	30 (57.69)	97 (72.93)		
History of lactational mastitis	Yes	16 (30.77)	43 (32.33)	0.838	0.838
	No	36 (69.23)	90 (67.67)		
History of hyperprolactinemia	Yes	15 (28.85)	40 (30.08)	0.869	0.869
	No	37 (71.15)	93 (69.92)		
History of breast trauma	Yes	11 (21.15)	24 (18.05)	0.628	0.628
	No	41 (78.85)	109 (81.95)		
Abscess formation	Yes	40 (76.92)	101 (75.94)	0.888	0.888
	No	12 (23.08)	32 (24.06)		
Sinus tract formation	Yes	10 (19.23)	21 (15.79)	0.573	0.573
	No	42 (80.77)	112 (84.21)		
History of hormone therapy	Yes	6 (11.54)	11 (8.27)	0.489	0.489
	No	46 (88.46)	122 (91.73)		
SII		200.01 ± 23.30	130.32 ± 27.79	16.010	<0.001
PCT (ng/L)		0.60 ± 0.04	0.59 ± 0.05	1.289	0.199
CRP (mg/L)		26.95 ± 2.04	21.81 ± 4.07	8.680	<0.001

IL-8, interleukin-8; PCT, procalcitonin; IL-6, interleukin-6; CRP, C-reactive protein.

### 3.3 Binary logistics regression analysis

Using overweight/obesity, SII, and CRP as independent variables, they were assigned values as shown in Table 3, a binary logistics regression analysis was conducted with therapeutic efficacy (poor = 1, good = 0) as the dependent variable. The results of the binary logistics regression analysis indicated that SII and CRP are influencing factors of therapeutic efficacy in NLM (*p* < 0.05), as shown in Table 4.

**Table 3:** Variable assignment.

Influencing factors	Assignment
SII	Original value
CRP	Original value
Overweight/Obesity	No = 0, Yes = 1

**Table 4:** Binary logistics regression analysis results.

Variable	*B*	SE	Wald	*p*	Exp (*B*)	95% CI	
						Lower limit	Upper limit
SII	0.823	0.188	19.221	<0.001	2.278	1.576	3.291
CRP	0.589	0.143	17.065	<0.001	1.802	1.363	2.383
Overweight/Obesity	0.723	0.685	1.116	0.291	2.061	0.539	7.888
Constant	−74.457	13.845	28.922	<0.001	<0.001	—	—

### 3.4 Construction of prediction model

**Figure 2:**
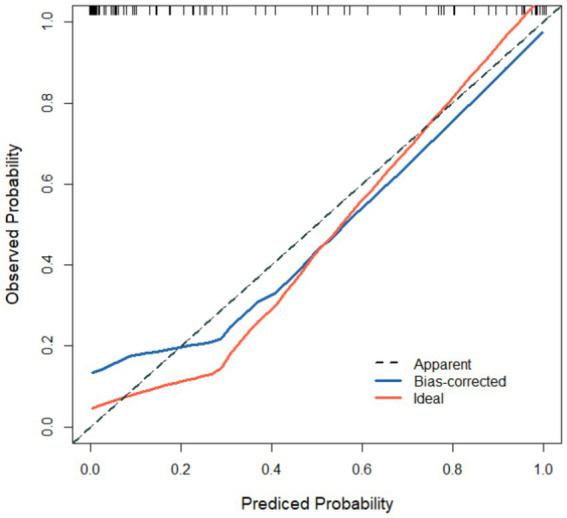
Calibration curve in modeling group. The curve is a line close to 1, indicating that the model predicts risk with good consistency with the actual risk.

**Figure 3:**
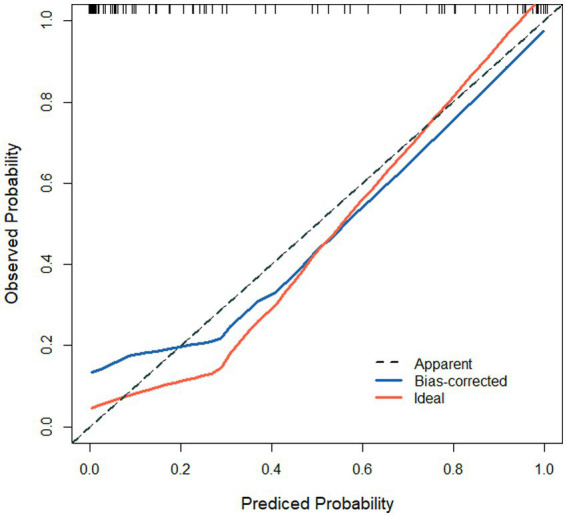
Calibration curve in validation group. The curve is a line close to 1, indicating that the model predicts risk with good consistency with the actual risk.

Based on the results of the logistic regression analysis, the variables SII and CRP (named X_1_ and X_2_, respectively) were included in the constructed prediction model. The expression of the combined detection factor model was determined as Logit(P) = −74.457 + (0.823X_1_) + (0.589X_2_). The calibration curve slope of the model in both the modeling group and validation group approximated a straight line, indicating good consistency between the predicted risk and actual risk, as shown in Figures 2, 3.

### 3.5 ROC curve

**Figure 4:**
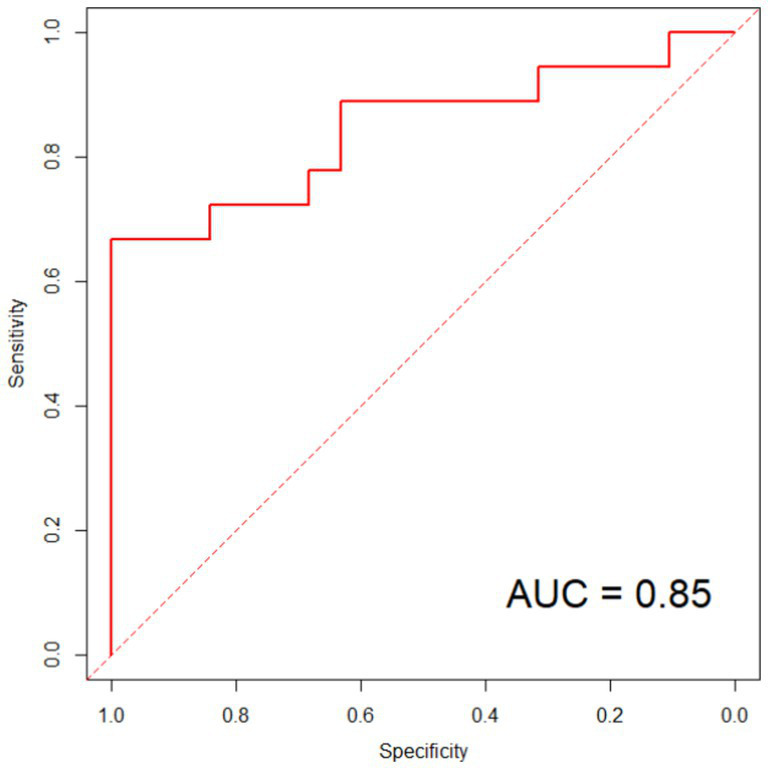
ROC curve in modeling group. The ROC curve has an area close to 1, indicating excellent predictive performance of the model in the modeling group.

**Figure 5:**
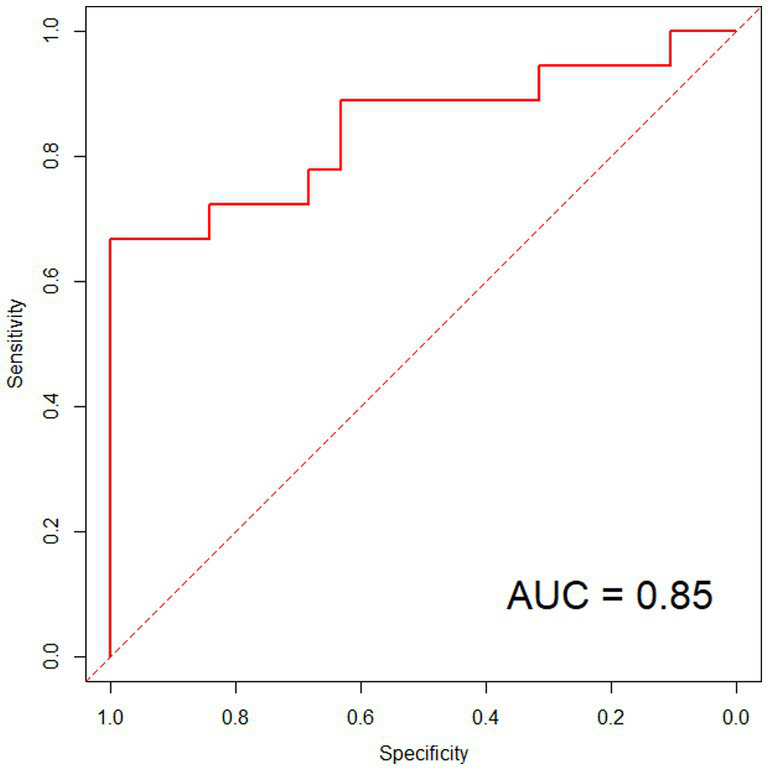
ROC curve in validation group. With an ROC curve of 0.7 < AUC ≤ 0.9, it indicates that the model has good predictive value in the validation group.

The ROC analysis results showed that, in the modeling group, the model’s predicted area under the curve was 0.94, with a standard error of 0.021 (95% CI: 0.823–0.966) and a Youden index of 0.80. At that time, the sensitivity was 88.84%, and the specificity was 90.73%, as shown in Figure 4. In the validation group, the model’s predicted area under the curve was 0.85, with a standard error of 0.037 (95% CI: 0.794–0.893) and a Youden index of 0.68. At that point, the sensitivity was 87.62%, and the specificity was 80.47%, as shown in Figure 5.

### 3.6 Clinical benefit analysis of prediction model

**Figure 6:**
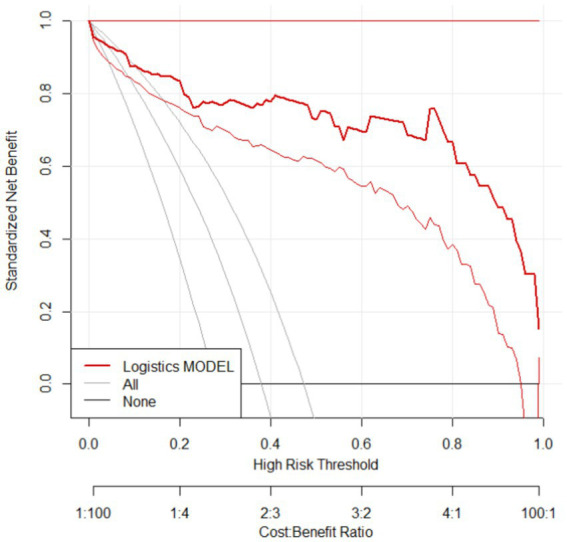
DCA curve in modeling group.

**Figure 7:**
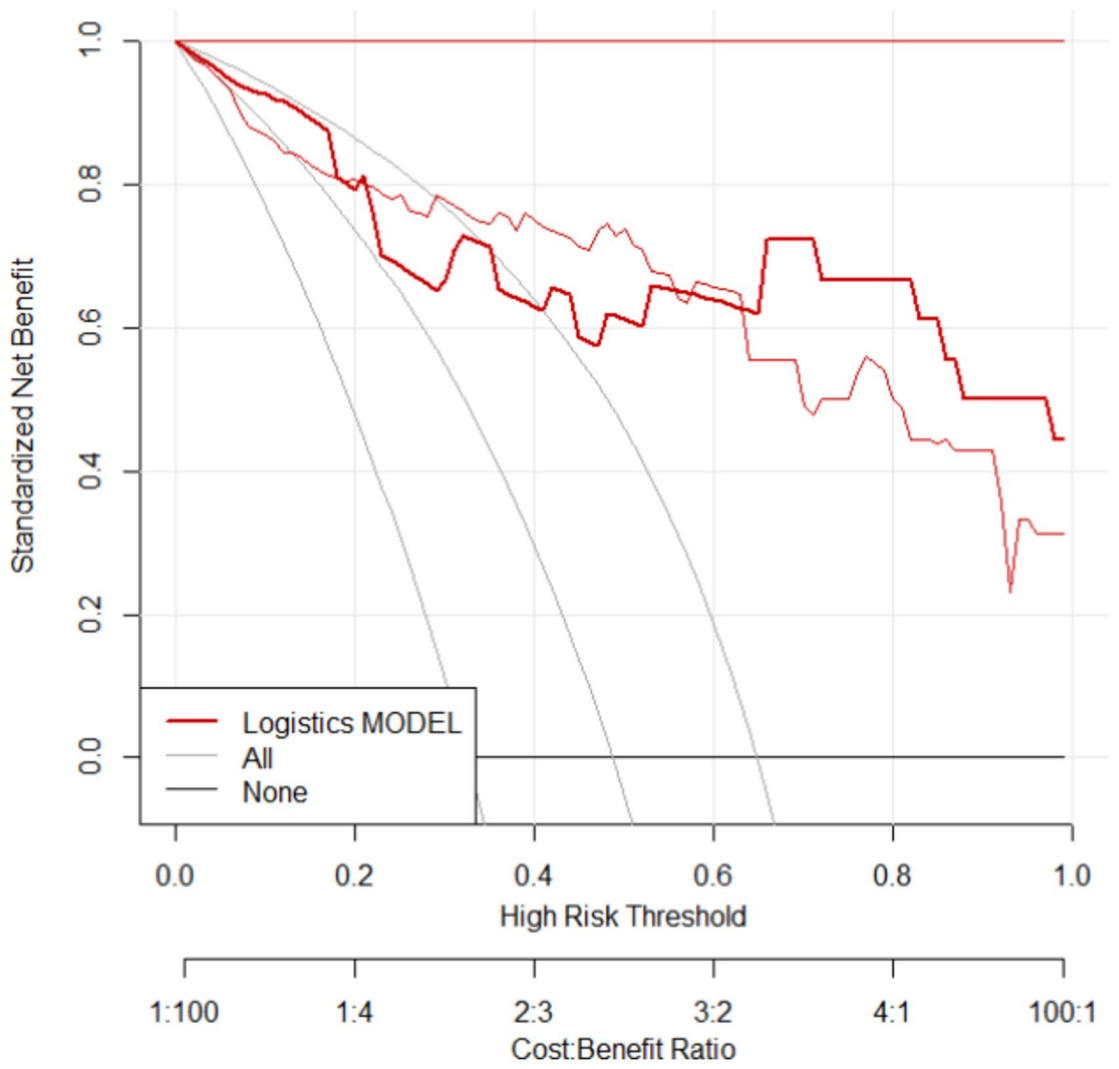
DCA curve in validation group.

Drawing DCA curve to evaluate the clinical utility of the model in predicting therapeutic efficacy, it is evident that the model exhibits a significant positive net benefit, indicating good clinical utility, as shown in Figure 6 (see Figure 7).

## 4 Discussion

In this study, we systematically analyzed the influencing factors of therapeutic efficacy in NLM. For the first time, the focus was directed towards hematological inflammatory indicators, particularly SII and CRP, which play crucial roles in inflammatory responses and immune regulation (2, 9). This study established a predictive model linking these hematological inflammatory indicators with the efficacy of NLM treatment. It provides a new perspective for understanding the pathogenesis of NLM and treatment outcome differences, laying a solid foundation for developing predictive models based on hematological inflammatory indicators. The establishment of this model aims to assist physicians in more accurately evaluating patient efficacy and prognosis, as well as providing a scientific basis for developing personalized treatment plans, thus improving the effectiveness of NLM treatment.

Through both univariate and binary logistics regression analyses in this study, SII and CRP were identified as independent influencing factors of therapeutic efficacy in NLM. Additionally, the findings of Huang et al. (10) indicate that CRP levels can serve as a biomarker for assessing the severity of IGM diseases, which aligns closely with the results of this study. SII and CRP, as crucial inflammatory factors, play significant roles in inflammatory responses and immune regulation, with their levels directly reflecting the body’s immune status and degree of inflammation (11). SII comprehensively reflects the body’s immune and inflammatory status. Elevated SII may indicate a strong inflammatory response, immune dysfunction, affecting disease progression and treatment response, thus influencing the efficacy of NLM treatment (12, 13). The results of Fetherston et al. (14) also demonstrate a significant increase in CRP levels in the blood during mastitis, further confirming the findings of this study.

Based on the results of the logistic regression analysis, the variables SII and CRP (named X_1_ and X_2_, respectively) were included in the constructed prediction model. The expression of the combined detection factor model was determined as Logit(P) = −74.457 + (0.823X_1_) + (0.589X_2_). Constant terms are the result of statistical calculations. In practical applications, the rationality of the prediction probability should be combined with the normal clinical range of variables (e.g., normal reference values for SII and normal thresholds for C-reactive protein). The calibration curve slopes of the model in the modeling group and validation group are both close to 1, indicating good consistency between the predicted risk and actual risk. ROC analysis results show that the model has high AUC in both the modeling and validation groups, with good sensitivity and specificity. Further evaluation of the clinical utility of the model in predicting therapeutic efficacy through DCA curve plotting reveals a significant positive net benefit, indicating the model’s good clinical utility and accuracy. This result indicates that the predictive model we have established can effectively predict the efficacy of NLM, providing strong support for clinical decision-making.

The predictive model established in this study holds significant clinical importance and practical value. Traditional examinations often have limited understanding of diseases through methods like ultrasound (15, 16), whereas this model can assist physicians in better assessing the condition of patients with NLM and treatment outcomes, thereby developing more personalized treatment plans. By detecting and analyzing hematological inflammatory indicators in patients, physicians can predict the effectiveness of treatment, adjust treatment plans promptly, and enhance treatment outcomes. Additionally, the model can provide essential references for prognostic evaluations of patients with NLM. By monitoring and analyzing inflammatory indicators of patients, physicians can promptly comprehend changes in the patient’s condition, assess prognostic risks, and take more effective intervention measures to reduce recurrence rates and the incidence of complications. Moreover, the model can offer new insights and methods for clinical research on NLM. Through in-depth analysis of the factors in the model, further exploration of the pathogenesis and immune regulation mechanisms of NLM can be conducted, providing theoretical support for the innovation of new drug development and treatment methods.

While this study has achieved certain results, there are still some limitations. Firstly, this study is a retrospective study with a relatively small sample size, which may lead to some bias in the results. Future research should involve multicenter, large-sample prospective studies to further validate the accuracy and reliability of the model. Secondly, this study primarily focused on hematological inflammatory indicators as predictive factors, neglecting other potential factors that may influence efficacy, such as genetic polymorphisms, environmental factors, etc. This study has several limitations. First, certain markers such as IL-6 were excluded due to missing data and lack of significance in pre-analysis, which may have resulted in the loss of important inflammatory information and affected the comprehensiveness of the model. Second, although overweight/obesity showed a correlation with therapeutic efficacy in univariate analysis, this association was not significant in multivariate regression, and the potential influence of confounding factors such as the severity of inflammation was not examined in depth. Stratified analyses were also not performed. Third, the relatively limited sample size may introduce selection bias and reduce the generalizability of the results. Finally, dynamic changes in SII were not monitored, making it difficult to evaluate its potential role in predicting treatment efficacy during disease progression. Future studies with larger sample sizes, more refined analyses, and dynamic monitoring are needed to validate and extend our findings. Future studies could delve into these factors to enhance the predictive model regarding the efficacy of NLM. Additionally, the predictive model established in this study is mainly applicable to NLM patients treated at our hospital, and its applicability and generalizability need further validation. Future researches could verify and optimize the model for NLM patients from different regions and populations to enhance its universality and accuracy.

## 5 Conclusion

In conclusion, this study successfully established a predictive model for the therapeutic efficacy of NLM based on hematological inflammatory indicators, providing robust support for clinical decision-making. Future researches can further explore other influencing factors on efficacy, improve the predictive model, and conduct multicenter, large-sample prospective studies to validate the accuracy and reliability of the model. Additionally, there is a need to enhance clinical research on NLM, delve into its pathogenesis and immune regulation mechanisms, and provide theoretical support for the innovation of new drug development and treatment methods.

## Data Availability

The raw data supporting the conclusions of this article will be made available by the authors, without undue reservation.
