# Correction: Factors influencing therapeutic efficacy of non-lactational mastitis based on hematological inflammatory markers and establishment of a predictive model

**DOI:** 10.3389/fmed.2026.1789363

**Published:** 2026-02-20

**Authors:** Jinjuan Peng, Li Li, Lili Fan, Qun Lu

**Affiliations:** 1 Department of Breast Surgery, The Fourth People's Hospital of Zhenjiang, The Fourth Affiliated Hospital of Jiangsu University, Zhenjiang, China; 2 Department of Breast Surgery, Changzhou Maternal and Child Health Care Hospital, Changzhou, China

**Keywords:** inflammatory markers, non-lactational mastitis, mastitis, SII, CRP

Affiliation “Department of Breast Surgery, The Fourth People's Hospital of Zhenjiang, The Fourth Affiliated Hospital of Jiangsu University, Zhenjiang, China” was erroneously given as “Department of Breast Surgery, The Fourth Affiliated Hospital of Jiangsu University, Zhenjiang, China.”

There was a mistake in Table 2 as published. There were data errors and missing values in the Good efficacy group (*n* = 133) column in Table 2.

The corrected Table 2 appears below.

**Table 2:** Univariate analysis of factors influencing therapeutic efficiency of NLM.

**Baseline data**		**Poor efficacy group (*n* = 52)**	**Good efficacy group (*n* = 133)**	***t*/χ^2^ Value**	***p-*Value**
Age (years)		35.96 ± 5.46	35.23 ± 5.40	0.824	0.411
Number of pregnancies	< 2	31 (59.62)	75 (56.39)	0.159	0.690
	≥2	21 (40.38)	58 (43.61)		
Menstrual disorders	Yes	33 (63.46)	85 (63.91)	0.003	0.955
	No	19 (36.54)	48 (36.09)		
Overweight/obesity	Yes	22 (42.31)	36 (27.07)	4.034	0.045
	No	30 (57.69)	97 (72.93)		
History of lactational mastitis	Yes	16 (30.77)	43 (32.33)	0.042	0.838
	No	36 (69.23)	90 (67.67)		
History of hyperprolactinemia	Yes	15 (28.85)	40 (30.08)	0.027	0.869
	No	37 (71.15)	93 (69.92)		
History of breast trauma	Yes	11 (21.15)	24 (18.05)	0.236	0.628
	No	41 (78.85)	109 (81.95)		
Abscess formation	Yes	40 (76.92)	101 (75.94)	0.020	0.888
	No	12 (23.08)	32 (24.06)		
Sinus tract formation	Yes	10 (19.23)	21 (15.79)	0.317	0.573
	No	42 (80.77)	112 (84.21)		
History of hormone therapy	Yes	6 (11.54)	11 (8.27)	0.478	0.489
	No	46 (88.46)	122 (91.73)		
SII		200.01 ± 23.30	130.32 ± 27.79	16.010	< 0.001
PCT (ng/L)		0.60 ± 0.04	0.59 ± 0.05	1.289	0.199
CRP (mg/L)		26.95 ± 2.04	21.81 ± 4.07	8.680	< 0.001

IL-8, interleukin-8; PCT, procalcitonin; IL-6, interleukin-6; CRP, C-reactive protein.

The original version of this article has been updated.

